# Dual-Level Ureteral Obstruction in Children: A Systematic Review Highlighting Diagnostic Challenges and Optimal Surgical Strategy

**DOI:** 10.3390/children13020305

**Published:** 2026-02-22

**Authors:** Olivia-Oana Stanciu, Andreea Moga, Radu Balanescu, Mircea Andriescu

**Affiliations:** 1 Department of Paediatric Surgery and Orthopaedics, “Carol Davila” University of Medicine and Pharmacy, 050474 Bucharest, Romania; olivia.stanciu@drd.umfcd.ro (O.-O.S.); radu.balanescu@umfcd.ro (R.B.); mircea.andriescu@umfcd.ro (M.A.); 2 Paediatric Surgery Department, “Grigore Alexandrescu” Clinical Emergency Hospital for Children, 011743 Bucharest, Romania

**Keywords:** UPJ obstruction, UVJ obstruction, megaureter, hydronephrosis, pediatric urology, pyeloplasty, ureteral reimplantation, PRISMA

## Abstract

**Highlights:**

**What are the main findings?**

- Preoperative identification of ipsilateral concomitant UPJ and UVJ obstruction in children is uncommon.
- Standard imaging modalities frequently fail to detect distal obstruction.
- Contrast pyelography improves diagnostic recognition of dual-level obstruction.
- A staged surgical approach beginning with pyeloplasty is commonly associated with favorable outcomes.

**What are the implications?**

- Heightened clinical suspicion is required in children with severe or persistent hydronephrosis.
- Intraoperative difficulty during ureteral stent placement should prompt reassessment for distal obstruction.
- Proximal-first surgical correction may reduce unnecessary distal ureteral reconstruction.
- Early diagnosis and appropriate surgical sequencing may help preserve renal function.

**Abstract:**

**Background:** Ipsilateral concomitant ureteropelvic junction (UPJ) and ureterovesical junction (UVJ) obstruction is an uncommon but clinically important pediatric condition. Because standard imaging often detects only one level of obstruction, the coexistence of both lesions is frequently overlooked. Delayed diagnosis may result in persistent hydronephrosis, recurrent urinary tract infections, and progressive renal injury. This systematic review synthesizes current evidence regarding diagnostic challenges, management strategies, and outcomes in children with dual UPJ–UVJ obstruction. **Methods:** A systematic review following PRISMA 2020 guidelines was conducted and prospectively registered in PROSPERO. Major databases were searched for studies describing pediatric patients with confirmed ipsilateral UPJ + UVJ obstruction. Extracted data included clinical presentation, diagnostic pathways, imaging modalities, timing of diagnosis, surgical sequencing, and postoperative outcomes. **Results:** Across the 8 included studies, preoperative recognition of dual obstruction was uncommon. Most cases were diagnosed intraoperatively when retrograde stent passage failed or postoperatively when hydronephrosis persisted after an apparently adequate first procedure. Retrograde or antegrade pyelography consistently outperformed ultrasonography and diuretic renography in identifying distal pathology. Staged repair—typically beginning with pyeloplasty—emerged as the most reliable approach, as correction of the proximal obstruction alone frequently improved distal drainage. UVJ-first strategies were less effective and often required secondary pyeloplasty. Endoscopic and minimally invasive techniques showed promise in selected patients but were reported in limited numbers with short follow-up. Functional renal outcomes generally stabilized or improved following complete correction, particularly when intervention occurred early in life. **Conclusions:** Dual UPJ–UVJ obstruction remains a diagnostic challenge in pediatric urology. Complementing standard imaging with contrast pyelography and maintaining vigilance during intraoperative stent placement can improve detection. Available reports suggest that a staged proximal-first surgical strategy can optimize drainage and reduce the risk of unnecessary distal reconstruction. Early intervention appears beneficial for renal recovery, though long-term outcomes remain insufficiently studied. Ongoing follow-up is essential, particularly in children with recurrent urinary tract infections or persistent hydronephrosis.

## 1. Introduction

Ureteropelvic junction obstruction and ureterovesical junction obstruction represent the two most common sites of congenital ureteral obstruction in children [1]. Although these conditions are well-characterized when occurring in isolation, their concomitant presentation on the same ipsilateral ureter is rare [2]. This dual obstruction represents a diagnostically challenging entity that has received limited systematic investigation.

Optimal management strategies remain controversial, particularly regarding the sequencing of surgical interventions and the role of minimally invasive techniques. Although outcomes for isolated UPJ or UVJ obstruction are well documented, evidence for dual obstruction is limited to small case series and individual reports [3,4]. While success rates exceed 95% for isolated UPJ or UVJ obstruction, outcomes data for dual obstruction remain limited to small case series and individual case reports.

To date, no comprehensive systematic review has synthesized the available evidence on diagnostic approaches, management strategies, and clinical outcomes in children with ipsilateral concomitant UPJ and UVJ obstruction.

Our primary objectives were to evaluate diagnostic strategies and their accuracy; compare management approaches, including simultaneous versus staged repairs; and assess clinical outcomes including surgical success, complications, and renal function preservation.

This review follows PRISMA guidelines and was prospectively registered with PROSPERO (registration number: CRD420261281285).

## 2. Materials and Methods

### 2.1. Study Design and Registration

This study followed the Preferred Reporting Items for Systematic Reviews and Meta-Analyses (PRISMA 2020) guidelines. The review protocol was prospectively registered in PROSPERO (ID: CRD420261281285). The objective was to synthesize available evidence on pediatric patients with ipsilateral concomitant ureteropelvic junction (UPJ) and ureterovesical junction (UVJ) obstruction, focusing on diagnostic pathways, management strategies, and clinical outcomes.

### 2.2. Search Strategy

Information sources included PubMed/MEDLINE, Embase, Scopus, Web of Science, and the Cochrane Library, which were searched from database inception to December 2024. Search terms combined controlled vocabulary and free-text keywords related to both levels of obstruction, including the following: “ureteropelvic junction obstruction”; “ureterovesical junction obstruction”; “primary obstructive megaureter”; “concomitant obstruction”; “dual obstruction”; “pediatric” OR “child”. Boolean combinations included (UPJ OR ureteropelvic) AND (UVJ OR ureterovesical OR megaureter) AND (child OR pediatric). Reference lists of eligible articles and relevant reviews were screened manually to ensure completeness. No language or date restrictions were applied during the initial search; however, only English-language articles were included at final selection. Manual reference screening did not identify additional eligible studies beyond those retrieved from database searches.

The full electronic search strategy for all databases is provided as the Supplementary Materials.

### 2.3. Eligibility Criteria

Eligibility criteria were defined according to a PICO framework.

Population: pediatric patients (≤18 years) with ureteral obstruction.

Intervention/Exposure: confirmed ipsilateral coexistence of ureteropelvic junction (UPJ) and ureterovesical junction (UVJ) obstruction.

Comparison: not applicable, as most included studies were observational without control groups.

Outcomes: diagnostic methods, management strategies, and postoperative or renal outcomes.

Studies were included if they reported pediatric patients with confirmed ipsilateral coexistence of UPJ and UVJ obstruction, with diagnosis established by imaging, intraoperative findings, or postoperative confirmation. Eligible study designs included retrospective or prospective cohort studies, case series with at least two patients, and mixed observational studies. Only English-language articles were included at the final selection stage.

Studies were excluded if they reported isolated UPJ or isolated UVJ obstruction, bilateral obstruction without side-specific analysis, neurogenic bladder, ureterocele, duplication anomalies, or vesicoureteral reflux without obstruction. Conference abstracts without extractable data and cases of secondary obstruction due to stones, tumors, or iatrogenic causes were also excluded. Single-patient case reports were eligible due to rarity, but were analyzed descriptively and not used to support comparative conclusions.

### 2.4. Study Selection Process

Two reviewers independently screened titles and abstracts, followed by full-text assessment. Discrepancies were resolved through discussion or consultation with a third reviewer. The PRISMA flow diagram was updated to reflect: corrected number of records retrieved; duplicate removal; studies excluded due to non-confirmatory diagnoses; final inclusion of eligible studies.

Only studies that clearly documented ipsilateral, anatomically or functionally confirmed dual obstruction were retained.

### 2.5. Data Extraction

Using a standardized form, two reviewers independently extracted data on the following: study characteristics (country, design, sample size); patient demographics; clinical presentation including UTI history when available; imaging modalities and diagnostic sequence; timing of diagnosis (preoperative, intraoperative, postoperative); operative strategy (UPJ-first, UVJ-first, simultaneous, endoscopic); postoperative course and need for secondary procedures; renal function trends; complications and follow-up duration.

Any disagreements were resolved by consensus.

### 2.6. Risk of Bias Assessment

Risk of bias was assessed using the Joanna Briggs Institute (JBI) critical appraisal tools appropriate to each study design. The JBI checklist for case series was used for retrospective case series, the JBI checklist for cohort studies was applied to retrospective cohort studies, and the JBI checklist for case reports was used for the single-patient report. The overall certainty of evidence was assessed qualitatively due to heterogeneity and the absence of comparative trials.

### 2.7. Data Synthesis and Analysis

Due to clinical and methodological heterogeneity—differences in diagnostic protocols, surgical techniques, definitions of obstruction, and follow-up duration—a quantitative meta-analysis was not feasible. Therefore, a narrative synthesis was performed, organizing findings into thematic domains: diagnostic pathways and their accuracy; timing and patterns of diagnosis; management strategies and surgical sequencing; clinical and renal outcomes; complications and safety considerations; relationship with recurrent urinary tract infections. Where possible, consistent trends across studies were summarized descriptively.

Given the rarity of dual-level obstruction, case reports were included to capture diagnostic and technical details; however, they were interpreted cautiously and summarized separately without implying comparative effectiveness.

## 3. Results

### 3.1. Study Selection

**Figure 1:**
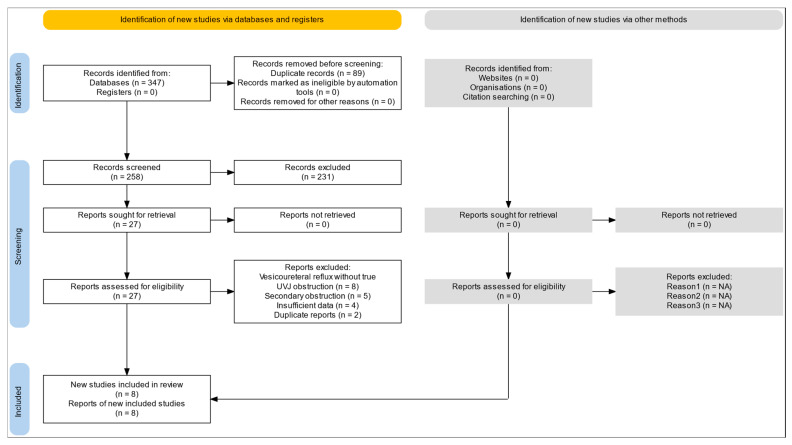
PRISMA 2020 flow diagram showing the selection process for studies on ipsilateral concomitant UPJ and UVJ obstruction in children.

The systematic search identified 347 potentially relevant citations across all databases (PubMed: 124, Embase: 98, Cochrane Library: 23, Web of Science: 67, Scopus: 35). After removing 89 duplicates, 258 records underwent title and abstract screening. Of these, 231 were excluded as they did not meet eligibility criteria (isolated UPJ or UVJ obstruction: 187; adult population only: 28; review articles: 16). Twenty-seven full-text articles were retrieved and assessed for eligibility. Nineteen articles were subsequently excluded (vesicoureteral reflux without true UVJ obstruction: 8; secondary obstruction: 5; insufficient data: 4; duplicate reports: 2). Ultimately, 8 observational studies reporting pediatric patients with ipsilateral concomitant UPJ and UVJ obstruction were included (Table 1). The PRISMA flow diagram is presented in Figure 1. Manual reference screening did not yield additional eligible studies.

Due to substantial clinical and methodological heterogeneity (different diagnostic criteria, diverse surgical approaches, variable follow-up protocols, and predominantly retrospective designs), meta-analysis was not feasible. Results are therefore presented as a narrative synthesis with structured data extraction tables.

Several included studies reported outcomes per renal unit rather than per individual patient. Consequently, reliable aggregation of a total patient number was not feasible, and results are presented descriptively at the study level.

### 3.2. Study Characteristics

A total of eight studies met the inclusion criteria. These consisted of five retrospective case series, two retrospective cohort studies, and one single-patient case report, published between 1987 and 2024. Together, they provide the most complete available evidence regarding ipsilateral concomitant ureteropelvic junction obstruction and ureterovesical junction/vesicoureteral junction/primary obstructive megaureter obstruction in children.

Study sizes varied significantly, ranging from single-patient case reports to larger series. The largest pediatric cohort was reported by Ebadi et al. (2013) [5], which included 47 renal units with concomitant proximal and distal obstruction identified among children treated for primary obstructive megaureter. Three additional studies provided medium-sized cohorts: McGrath (14 cases) [6], Pesce (11 cases) [7], Cay (14 cases) [8], and Lee (15 cases) [9]. Smaller series included Halder (5 cases) [10] and Moodley (2 cases) [2], whereas Di Fabrizio (2024) [11] contributed one pediatric case treated with a combined minimally invasive strategy. Study size and reporting units varied considerably, with some cohorts reporting outcomes per patient and others per renal unit, reflecting differences in study design and surgical focus.

Age at presentation was generally early, most frequently in infancy or early childhood, reflecting congenital etiologies. Across studies, boys were more commonly affected, though exact sex distribution could not be reliably extracted from several older publications. Laterality was inconsistently reported; only Di Fabrizio explicitly documented a right-sided obstruction, while the others did not systematically specify side involvement.

All included studies confirmed true anatomic dual obstruction rather than functional or secondary obstruction. Confirmation modalities included a combination of ultrasonography, diuretic renography (MAG3/DTPA), retrograde pyelography, antegrade pyelography, or intraoperative assessment (particularly failure to pass a catheter or stent across the UVJ).

Management strategies varied widely by center, era, and surgeon preference, including pyeloplasty alone (with secondary UVJ surgery if persistent), ureteral reimplantation alone, simultaneous UPJ + UVJ repair, endoscopic balloon dilation (Ebadi cohort), staged repair (UPJ-first or UVJ-first), and minimally invasive combined procedures (Di Fabrizio) [11].

Outcome definitions and follow-up duration also varied, limiting comparability and preventing meta-analysis. Renal function improvement, postoperative resolution of hydronephrosis, and need for reoperation were the most consistently reported metrics.

**Table 1:** Characteristics of Included Studies.

Study	Country	Study Type	Reported Sample Size	Confirmation of Dual Obstruction	Age Range
McGrath 1987 [6]	USA	Retrospective series	14	IVP, US, intraop findings	Not specified
Pesce 2003 [7]	Italy	Retrospective series	11	US, renography, RUPG	1 mo–9 yrs
Cay 2006 [8]	Turkey	Retrospective series	14	US, renography, intraop	2 mo–6 yrs
Moodley 2010 [2]	South Africa	Case series	2	Retrograde pyelography	2–4 yrs
Ebadi 2013 [5]	Iran	Large retrospective cohort	47 renal units	Intraop + post-pyeloplasty imaging	1–12 yrs
Lee 2014 [9]	Korea	Retrospective cohort	15	US, renography, RP/AGP, intraop	3 mo–8 yrs
Halder 2014 [10]	India	Retrospective series	5	US, renography, intraop	Neonate–3 yrs
Di Fabrizio 2024 [11]	Italy	Case report	1	MRU + cystoscopy + intraop	14 yrs

Sample size is reported as described in the original studies; some cohorts reported outcomes per renal unit rather than per individual patient.

### 3.3. Risk of Bias Assessment

Overall, the methodological quality of the included studies was limited, primarily due to their retrospective nature, small sample sizes, and incomplete reporting—an inherent challenge in research on this rare condition. All eight studies were observational and lacked control groups, randomization, or blinding. Consequently, risk of bias across domains was generally rated as moderate to high. Cohort studies and the single case report were appraised using their respective JBI tools.

Selection bias was present in most studies, as cases were identified through surgical or radiological databases rather than through prospective enrollment. Three studies (McGrath, Moodley, Halder) [2,6,10] were small case series in which patient selection could not be independently verified. Reporting bias was also a concern; laterality, sex distribution, precise diagnostic criteria, and follow-up duration were inconsistently reported. Only the more recent cohorts (Ebadi and Lee) [5,9] presented clearer methodological descriptions and explicit diagnostic confirmation pathways.

Outcome assessment bias was likely significant, as most studies relied on imaging interpretation without standardized criteria for defining obstruction resolution or treatment success. Furthermore, follow-up intervals varied widely, and postoperative functional outcomes such as differential renal function were not uniformly assessed. No study used validated scoring systems or predefined thresholds for intervention or reoperation.

Despite these limitations, the studies consistently confirmed the presence of true anatomic obstruction at both the UPJ and UVJ/POM levels, strengthening internal validity regarding diagnostic confirmation and anatomical characterization. However, heterogeneity and incomplete data precluded meta-analysis or statistical pooling. Given these limitations, the findings of this review should be interpreted as hypothesis-generating rather than definitive.

### 3.4. Diagnostic Challenges and Timing of Diagnosis

All included studies reported substantial difficulty in preoperative identification of ipsilateral concomitant ureteropelvic junction (UPJ) and ureterovesical junction (UVJ) obstruction. Across the reviewed literature, dual obstruction was most frequently recognized either intraoperatively or after persistence of hydronephrosis following initial surgical correction of a presumed isolated obstruction.

Preoperative diagnosis of both obstruction levels was uncommon. In several studies, routine imaging modalities such as ultrasonography and diuretic renography identified only a single level of obstruction, most often the proximal UPJ. Distal UVJ obstruction was frequently not detected prior to surgery and became apparent only during operative assessment or postoperative follow-up.

Intraoperative identification occurred primarily during attempts at retrograde stent or catheter placement, when passage across the ureterovesical junction was not possible, prompting further evaluation. This pattern was reported consistently across multiple series, regardless of publication period.

Postoperative recognition represented another common diagnostic scenario. Several studies described cases in which hydronephrosis or impaired drainage persisted after apparently adequate correction of one obstruction site, leading to additional investigations that subsequently confirmed the presence of a second, previously unrecognized obstruction.

Contrast-based imaging modalities played a key role in confirming dual obstruction. Retrograde pyelography was reported as particularly useful for delineating distal ureteral anatomy and identifying UVJ obstruction. Antegrade pyelography was less frequently employed but was utilized in selected cases, especially when postoperative drainage remained inadequate. In contrast, ultrasonography and diuretic renography alone were insufficient to reliably detect distal obstruction in a substantial proportion of cases.

Based on timing of recognition, three diagnostic patterns were consistently observed across studies: (1) preoperative diagnosis following contrast pyelographic evaluation; (2) intraoperative identification prompted by technical difficulty during stent placement; and (3) postoperative diagnosis after failure of initial surgical treatment to resolve hydronephrosis.

Diagnostic modalities are summarized in Table 2.

### 3.5. Management Strategies

Management strategies were heterogeneous and largely dependent on institutional preference, era of publication, and surgeon experience.

Management approaches for concomitant UPJ and UVJ obstruction varied considerably across the included studies, reflecting differences in institutional preferences, period of publication, and available surgical and endoscopic technologies. The strategies employed can be broadly grouped into UPJ-first staged repair, UVJ-first staged repair, simultaneous repair, and minimally invasive or endoscopic approaches.

#### 3.5.1. UPJ-First Staged Management

A UPJ-first approach was the most consistently applied strategy in the majority of series, including those of Pesce (2003) [7], Cay (2006) [8], Moodley (2010) [2], Halder (2014) [10], and Lee (2014) [9]. In these studies, pyeloplasty was performed as the initial intervention, followed by reassessment of distal drainage. Children who continued to demonstrate persistent ureteral dilatation or impaired emptying underwent subsequent distal ureteric reconstruction.

This approach was selected primarily because proximal obstruction was believed to exert a masking effect on distal function. Several authors emphasized that relieving the UPJ obstruction often allowed normalization of UVJ drainage, thereby avoiding unnecessary ureteral reimplantation. Pesce [7] and Cay [8] reported multiple cases in which initial pyeloplasty eliminated the need for UVJ intervention. Moodley [2] similarly observed that retrograde pyelography after pyeloplasty was essential in determining whether secondary UVJ repair was needed. Halder [10] documented a similar pattern, noting that postoperative imaging clarified whether distal obstruction persisted after proximal repair.

While the proportion of patients experiencing spontaneous improvement at the distal junction varied across studies, all authors agreed that UPJ-first repair was a safe initial strategy that minimized operative risk to the ureter’s vascular supply and allowed more accurate postoperative functional assessment.

#### 3.5.2. UVJ-First Staged Management

A UVJ-first approach was less commonly adopted. McGrath (1987) [6] included a subset of children who initially underwent distal ureteral reimplantation; however, all subsequently required pyeloplasty, indicating that addressing the UVJ obstruction alone did not resolve the proximal obstruction. This pattern highlighted the risk of treating a distal narrowing prematurely when a dominant UPJ obstruction may be the primary driver of pathology.

No included study demonstrated consistent improvement of UPJ obstruction following UVJ repair, reinforcing that a UVJ-first strategy is unlikely to be beneficial and may increase the likelihood of requiring multiple procedures.

#### 3.5.3. Simultaneous UPJ + UVJ Repair

Simultaneous repair was reported sparsely and with significant caution. McGrath [6] and Pesce [7] referenced isolated cases in which concurrent pyeloplasty and ureteral reimplantation were performed in a single session; however, concern for ureteral devascularization was raised, as both proximal and distal dissection increases the risk of compromising longitudinal blood supply.

One early case from the literature (referenced in multiple discussions) described ureteral ischemia leading to nephrectomy following simultaneous repair, reinforcing the need for caution. Due to these risks and lack of consistent long-term outcome data, simultaneous open repair was generally discouraged across most studies.

#### 3.5.4. Minimally Invasive and Endoscopic Approaches

Two studies contributed unique perspectives on less invasive management pathways:

Ebadi reported a large cohort of children with combined proximal and distal obstruction managed primarily with endoscopic balloon dilation [5]. Although this cohort was not exclusively composed of classic UPJ + UVJ obstruction cases, the subset with dual obstruction demonstrated high rates of improvement following endoscopic treatment. Repeat dilations were sometimes required, and some children ultimately progressed to open repair, but the overall experience indicated that endoscopic decompression could be an effective first-line option in selected cases.

Di Fabrizio described a novel combined strategy employing retroperitoneoscopic pyeloplasty together with cystoscopic balloon dilation of the distal ureter during the same anesthetic session [11]. This approach was successfully applied in a single pediatric patient, with no evidence of ureteral ischemia on follow-up. Although promising, this technique remains experimental, and larger clinical series are needed to validate safety and reproducibility.

#### 3.5.5. Summary of Management Patterns Across Studies

Across all included studies, several consistent themes emerged: UPJ-first repair was the most commonly used and most consistently effective strategy; UVJ-first repair was rarely successful as monotherapy and usually required subsequent pyeloplasty; simultaneous repair is technically feasible but carries higher vascular risk and limited outcome documentation; endoscopic and minimally invasive approaches show potential but require further validation before being widely adopted.

Overall, the evidence supports an initial UPJ-focused intervention followed by reassessment of distal function as a commonly adopted, practice-informed approach for managing concomitant UPJ and UVJ obstruction in children. Details are summarized in Table 3.

#### 3.5.6. Surgical Techniques Employed at the UPJ and UVJ

Detailed descriptions of the exact surgical techniques were inconsistently reported across the included studies, reflecting differences in reporting standards and publication eras. UPJ-level surgery was most commonly performed using pyeloplasty, usually through an open approach in earlier series. More recent studies described minimally invasive techniques, including laparoscopic or retroperitoneoscopic pyeloplasty. Endoscopic balloon dilation was reported in selected cases, particularly in the cohort described by Ebadi et al. UVJ-level surgery was generally performed by ureteral reimplantation when distal obstruction persisted after initial treatment. In some more recent reports, endoscopic balloon dilation of the distal ureter was also used.

Direct comparison of outcomes according to specific surgical techniques was not feasible. Most studies were small, retrospective, and heterogeneous, and several did not specify the exact operative methods. Across the literature, outcomes appeared to be influenced more by the sequencing of repair than by the specific technique used.

### 3.6. Clinical Outcomes

Outcome definitions and follow-up protocols varied substantially across studies, limiting direct comparison and precluding quantitative synthesis.

Clinical outcomes varied according to the management strategies employed and the timing of diagnosis. Across all included studies, successful resolution of hydronephrosis and symptomatic improvement were achievable in most children once both levels of obstruction were adequately treated. However, the heterogeneity of outcome definitions, imaging protocols, and follow-up duration limits direct quantitative comparison.

Outcomes across the literature can be summarized in four domains:

1. Resolution of hydronephrosis and drainage;
2. Need for secondary procedures;
3. Functional renal recovery;
4. Long-term results (where reported).

#### 3.6.1. Resolution of Hydronephrosis and Drainage

Most series reported satisfactory postoperative improvement in upper tract drainage following correction of one or both obstruction sites. In the UPJ-first cohorts (Pesce, Cay, Halder, Moodley) [2,7,8,10], a substantial proportion of children demonstrated improved distal drainage after pyeloplasty alone, indicating that some cases of UVJ narrowing were functional rather than fixed. Persistent distal obstruction, when present, became evident on follow-up ultrasonography or renography and was subsequently corrected with ureteral reimplantation or endoscopic management.

Lee’s cohort demonstrated improvement in renal pelvis dimensions and drainage curves in children undergoing pyeloplasty-first repair, with more variable outcomes in UVJ-first patients [9]. McGrath and Cay similarly reported favorable outcomes after staged surgical correction, though with variable time to stabilization [6,8].

In the endoscopic series (Ebadi), drainage normalized or significantly improved in most renal units following balloon dilation, with repeat dilation or secondary open repair required in a minority [5]. Di Fabrizio’s single-patient minimally invasive combined approach resulted in complete radiologic resolution [11].

#### 3.6.2. Need for Secondary Procedures

The need for reoperation varied among studies and was closely linked to the initial surgical strategy:

UPJ-first strategy:

Several studies (Pesce, Cay, Moodley, Halder, Lee) [2,7,8,10] documented that some children required subsequent UVJ repair if distal obstruction persisted after pyeloplasty. The proportion varied across cohorts but was consistently lower than in UVJ-first strategies.

UVJ-first strategy:

McGrath reported that all children who underwent initial distal reimplantation required subsequent pyeloplasty, demonstrating that treating the UVJ first did not prevent the need for proximal repair [6].

Endoscopic approaches:

In Ebadi’s cohort, some renal units required repeat dilation or conversion to open surgery; however, most responded to endoscopic intervention alone [5].

Simultaneous repair:

Due to limited use, reliable rates of secondary surgery cannot be estimated. Individual cases reported good outcomes, but concerns regarding ureteral vascularity persisted.

#### 3.6.3. Functional Renal Recovery

Only a minority of studies provided extractable functional renal outcome data. Among those that did, the patterns were consistent: renal function stabilized or improved following appropriate correction of both obstruction sites; children diagnosed and treated earlier in life demonstrated greater potential for functional recovery; long-standing obstruction was associated with less reversible loss of renal function.

Lee’s cohort presented the clearest functional trend: children with higher preoperative differential renal function experienced greater postoperative improvement, while those with severely reduced preoperative values exhibited limited recovery despite anatomically successful surgery [9].

Other series (Pesce, Cay, Halder) [7,8,10] reported qualitative improvement in drainage curves or renal pelvis size, though numerical differential renal function values were not consistently provided.

#### 3.6.4. Long-Term Outcomes and Follow-Up Duration

Long-term follow-up was inconsistently reported across studies. In earlier series (McGrath, Pesce, Cay) [6,7,8], follow-up ranged from months to a few years, with most children remaining asymptomatic and demonstrating stable imaging findings.

Ebadi’s endoscopic cohort provided longer follow-up intervals and demonstrated durable drainage improvement in most renal units, though some late failures were noted and required additional intervention [5].

Lee’s cohort offered structured follow-up into early childhood and showed stable outcomes after staged UPJ-first repair [9].

No study provided systematic follow-up beyond late childhood or adolescence. Di Fabrizio reported short-term success in a single case, but longer-term durability of combined minimally invasive repair remains unproven.

Summary of Clinical Outcomes (Narrative)

Across all included studies, hydronephrosis improved in most patients after correcting one or both obstructions; UPJ-first repair had the most predictable success and lowest reoperation burden; UVJ-first repair consistently failed to resolve proximal obstruction; endoscopic treatment showed high early success, with variable need for re-intervention; functional renal recovery depended strongly on preoperative renal reserve and age at intervention; long-term data remain limited; and no study tracked outcomes beyond adolescence.

## 4. Discussion

Ipsilateral concomitant ureteropelvic junction (UPJ) and ureterovesical junction (UVJ) obstruction represents a rare but clinically significant anomaly within the spectrum of congenital obstructive uropathies. Although individually common, their simultaneous presence on the same ureter is distinctly uncommon and remains poorly characterized in the pediatric literature. The studies included in this review consistently demonstrate that the coexistence of proximal and distal obstruction challenges traditional diagnostic algorithms and complicates surgical decision-making. Integrating these findings with broader pediatric urological principles highlights important implications for early recognition, rational sequencing of interventions, and long-term renal preservation.

### 4.1. Interpretation of Key Findings

While the primary literature describes each component obstruction in detail, the simultaneous occurrence has historically been underrecognized. Across nearly four decades of published experience, dual obstruction was rarely identified preoperatively [6,7,8,10].

This pattern indicates that dual obstruction differs clinically from isolated UPJ or UVJ obstruction. Proximal obstruction may obscure antegrade flow and thereby “mask” distal narrowing, while significant UVJ obstruction may produce a passive dilation of the renal pelvis that mimics UPJ obstruction. These intertwined dynamics explain why standard ultrasonography and renography often fail to localize pathology precisely. Rather than representing a failure of imaging technology, this reflects the inherent physiologic complexity of ureteral drainage under dual-level compromise.

The studies also collectively emphasize that initial surgical plans based on incomplete diagnosis often require modification. Children who underwent isolated repair of one level frequently exhibited persistent dilation postoperatively, prompting further intervention. This reinforces that careful preoperative evaluation should aim not only to confirm obstruction but also to exclude additional levels of obstruction.

### 4.2. Pathophysiological Integration and Theoretical Considerations

Although definitive mechanistic studies are lacking, a unifying pathophysiological theme emerges from the literature. Both the UPJ and UVJ are recognized transition zones where coordinated peristaltic activity is essential for antegrade flow [12,13]. Disruption of neuronal signaling, smooth muscle differentiation, or extracellular matrix organization could theoretically impair motility at both locations.

Although speculative, the broader CAKUT literature suggests that shared developmental or neuromuscular mechanisms may contribute to dual-level obstruction [14,15]. Even though genetic analyses specific to dual obstruction have not been reported, known associations with genes involved in ureteral peristalsis—such as BMP4, RET, and TSHZ3—suggest a plausible shared susceptibility [16,17]. Chronic obstruction at one level may also influence function at the other [18].

This pathophysiological interpretation helps explain why some distal obstructions improve after pyeloplasty, why simultaneous repair carries vascular risk, and why intraoperative findings may differ from preoperative imaging.

### 4.3. Diagnostic Implications and Evolving Strategies

The key diagnostic lesson emerging from the included studies is the essential role of contrast pyelography—particularly retrograde pyelography—in differentiating single from dual obstruction [2,9]. While ultrasonography and diuretic renography remain first-line modalities due to their noninvasive nature, their inability to resolve complex anatomy necessitates additional imaging when suspicion persists.

Retrograde pyelography allows the direct delineation of distal ureteral anatomy and obstruction. Antegrade pyelography, although less commonly used, is especially valuable postoperatively when hydronephrosis persists without clear explanation. Intraoperative stent passage failure was a highly informative clue in multiple studies and should be recognized as a diagnostic sign rather than a technical setback [6,8].

Advanced functional MR urography holds promise for noninvasive characterization of ureteral peristalsis and obstruction levels. However, published experience remains extremely limited, with no comparative studies to guide routine implementation.

### 4.4. Surgical Management: Rationale for a UPJ-First Strategy

When proximal and distal obstruction coexist, sequencing of surgical interventions becomes particularly important. Most included studies describe an initial UPJ-first staged approach, although the evidence is limited to retrospective series without comparative trials [7,8,9]:

1. Proximal obstruction may create functional distal impairment. Correction of proximal obstruction has been shown to improve distal drainage in several series.

2. UVJ-first repair does not relieve proximal obstruction. UVJ-first repair rarely obviated the need for subsequent pyeloplasty [6].

3. Simultaneous open repair risks ureteral vascular compromise. Mobilization at both ends of the ureter disrupts segmental blood supply, increasing the risk of ischemia. This concern, documented across several decades of literature, justifies a staged approach [7].

4. Staged surgery allows natural history observation. If distal drainage improves after pyeloplasty, unnecessary ureteral reimplantation can be avoided.

Endoscopic balloon dilation appears promising in selected cases, particularly where distal aperistalsis predominates. However, its long-term durability in true dual obstruction remains uncertain, and further comparative studies are needed. The specific surgical techniques used at each level were variably reported and largely reflected the era and institutional practice patterns. Most proximal repairs consisted of pyeloplasty, while distal obstruction was usually managed with ureteral reimplantation when required. Minimally invasive and endoscopic techniques were described in more recent series, but comparative outcome data were lacking. Overall, the sequencing of surgery—particularly a UPJ-first staged approach—appeared to influence outcomes more consistently than the specific operative technique.

### 4.5. Functional Outcomes: Interpretation and Clinical Implications

Quantitative renal function data were inconsistently reported across the included studies, making pooled analysis impossible. Nevertheless, several important qualitative patterns emerge: early intervention is associated with better functional recovery; kidneys with borderline function may stabilize even if full recovery is not achieved; persistent obstruction after the first procedure is a strong predictor of the need for secondary intervention.

These findings align with the broader obstructive uropathy literature, which emphasizes the importance of timely decompression to mitigate nephron loss, interstitial fibrosis, and long-term progression to chronic kidney disease [12,13,19]. Given the lifelong risk of hypertension and CKD in congenital obstructive uropathy, careful long-term follow-up is warranted even after apparently successful surgical correction.

### 4.6. Complications and Safety Considerations

Reported complications were infrequent but varied, reflecting differences in technique, institutional practice, and length of follow-up. The most clinically significant safety concern is the risk of ureteral devascularization when both ends of the ureter are mobilized extensively during simultaneous open repair [7]. This reinforces the rationale for staged reconstruction.

Postoperative urinary tract infections occurred in several cases but are a known risk in obstructed systems and not specific to dual obstruction. Endoscopic approaches demonstrated favorable short-term safety profiles but require structured follow-up due to the potential need for repeat treatment.

### 4.7. Relationship to Recurrent Urinary Tract Infections in Children

Recurrent urinary tract infections (UTIs) represent a significant clinical intersection between congenital obstructive uropathies and pediatric infectious disease. Children with ureteropelvic and ureterovesical junction obstruction—particularly when both levels of obstruction coexist—are predisposed to recurrent febrile UTIs due to impaired urinary drainage, increased intrapelvic pressure, and stasis that facilitates bacterial colonization. Multiple studies in the obstructive uropathy literature demonstrate that urinary stasis is one of the strongest predictors of recurrent infection, independent of vesicoureteral reflux. In the context of dual obstruction, this risk may be further amplified because proximal dilation alone does not fully decompress the collecting system when the distal ureter remains functionally or anatomically narrow. Consequently, these children frequently present with pyelonephritis prior to definitive diagnosis, and UTIs may be the first clinical manifestation of dual-level obstruction in otherwise asymptomatic infants.

Early recognition of dual obstruction has important implications for antibiotic stewardship and long-term prognosis. Children with persistent UTIs despite appropriate prophylaxis or with recurrent febrile episodes in the context of hydronephrosis should prompt clinicians to reassess for incomplete drainage, including the possibility of concomitant proximal and distal obstruction. This reinforces the need for close collaboration between pediatric nephrologists, infectious disease specialists, and pediatric urologists. Timely surgical correction not only improves drainage and renal function but also reduces the frequency and severity of UTIs, diminishing the cumulative risk of renal scarring. Thus, integrating the diagnostic and management principles from this review into UTI care pathways aligns with modern precision-medicine strategies: treating the underlying cause of infection rather than repeatedly managing its consequences.

### 4.8. Strengths and Limitations of the Evidence Base

The primary strength of this review is the comprehensive identification of all published cases of ipsilateral dual-level obstruction. The multicenter and multi-decade nature of the literature offers a broad perspective on diagnostic pitfalls and evolving management strategies.

However, the evidence base has substantial limitations: all studies were retrospective, small, and heterogeneous; there is no standardized diagnostic or surgical protocol; outcome definitions vary widely; follow-up is short and inconsistent; functional data are incomplete; long-term renal outcomes are largely unavailable. These limitations precluded quantitative synthesis and weaken the certainty of recommendations.

Inclusion of case reports increases susceptibility to publication bias and limits generalizability; therefore, findings were interpreted as practice-informed rather than evidence-based.

Laterality (left versus right side) was rarely reported across the included studies, preventing assessment of any side predominance or anatomical predisposition. Although such information could contribute to understanding developmental or anatomical patterns of obstruction, the available literature did not provide sufficient consistent reporting to support meaningful analysis. Future studies and registries should report laterality systematically to allow exploration of potential side-specific trends.

### 4.9. Clinical Implications

Based on the integrated interpretation of the literature, several practical points emerge for clinicians:

1. Maintain suspicion for dual obstruction in severe hydronephrosis or discordant imaging.
2. Use retrograde pyelography when the level of obstruction is unclear or when stent passage fails.
3. Prefer staged reconstruction, beginning with pyeloplasty.
4. Reassess distal function before proceeding to ureteral reimplantation.
5. Consider minimally invasive approaches selectively and with appropriate expertise.
6. Implement structured follow-up, as recurrence or persistence may manifest months after initial repair [12].

### 4.10. Future Research Directions

Given the rarity of this condition, meaningful progress will require the following: prospective multicenter registries; standardized diagnostic criteria; comparative studies of surgical sequencing; evaluation of MR urography and functional imaging; genetic and molecular research into ureteral dysmotility; long-term renal outcomes into adolescence and adulthood.

Such data would allow development of evidence-based clinical algorithms and may inform personalized approaches to management.

## 5. Conclusions

Ipsilateral concomitant ureteropelvic junction (UPJ) and ureterovesical junction (UVJ) obstruction in children is a rare but clinically significant condition associated with substantial diagnostic difficulty and potential risk to renal function. The available evidence, although limited and heterogeneous, highlights the consistently low rate of preoperative recognition and the frequent need for diagnostic reassessment during or after initial surgical management.

Heightened clinical suspicion and comprehensive diagnostic evaluation are essential in children with severe or persistent hydronephrosis. Adjunctive use of contrast pyelography, particularly when standard imaging is inconclusive or intraoperative stent passage is unsuccessful, plays a critical role in identifying dual-level obstruction. Current data support a staged surgical strategy favoring initial correction of the proximal obstruction, followed by reassessment of distal drainage, as this approach may optimize outcomes while avoiding unnecessary ureteral reconstruction.

Early diagnosis and appropriately sequenced intervention appear beneficial for renal preservation, whereas delayed recognition may limit functional recovery. However, long-term outcomes remain insufficiently characterized due to small sample sizes, retrospective study designs, and inconsistent reporting. Future multicenter collaboration and prospective data collection are needed to refine diagnostic algorithms and establish evidence-based management strategies for this uncommon but important pediatric condition.

## Figures and Tables

**Table 2:** Diagnostic Modalities Reported Across Included Studies.

Study	Ultrasound	Renogram (MAG3/DTPA)	Retrograde Pyelography	Antegrade Pyelography	Intraoperative Diagnosis
McGrath 1987 [6]	✔	✔	✔	✖	✔
Pesce 2003 [7]	✔	✔	✔	✖	✔
Cay 2006 [8]	✔	✔	✔	✖	✔
Moodley 2010 [2]	✔	✔	✔	✖	✔
Ebadi 2013 [5]	✔	✔	✖	occasionally	✔
Lee 2014 [9]	✔	✔	✔	✔	✔
Halder 2014 [10]	✔	✔	✔	✖	✔
Di Fabrizio 2024 [11]	✔	✔	✔	✖	✔

The presence of a diagnostic modality indicates that it was reported or utilized in the corresponding study. This table does not reflect systematic application, diagnostic accuracy, frequency of use, or comparative effectiveness between modalities.

**Table 3:** Management Strategies Reported in Included Studies.

Study	Initial Management	Additional Procedures	Staged or Simultaneous	Notes
McGrath 1987 [6]	UVJ-first in some, UPJ-first in others	High rate of secondary surgery	Mixed	UVJ-first approach was followed by subsequent pyeloplasty in all reported cases.
Pesce 2003 [7]	Pyeloplasty + later reimplantation	Yes	Staged	Staged repair was reported, with concern for ureteral vascular supply noted by the authors.
Cay 2006 [8]	Pyeloplasty	UVJ intervention in persistent cases	Staged	Emphasis on diagnostic difficulty.
Moodley 2010 [2]	Pyeloplasty	Reimplantation if needed	Staged	Retrograde pyelography was utilized for diagnostic clarification in the reported cases.
Ebadi 2013 [5]	Endoscopic balloon dilatation	Re-dilation or open repair	Primarily single-stage endoscopy	Endoscopic balloon dilation was reported as initial management, with repeat intervention or open surgery required in some cases.
Lee 2014 [9]	Pyeloplasty-first vs. UVJ-first	Yes	Both approaches compared	Pyeloplasty-first and UVJ-first strategies were both reported; secondary procedures were more frequently required following initial UVJ repair.
Halder 2014 [10]	Pyeloplasty or UVJ repair	Secondary procedures common	Staged	Diagnosis often intraoperative.
Di Fabrizio 2024 [11]	Combined minimally invasive	None	Simultaneous	Combined minimally invasive approach reported in a single pediatric case.

Management strategies are reported as described in the original studies. Direct comparison between approaches is limited by study heterogeneity, small sample sizes, and retrospective design.

## Data Availability

Data supporting the reported results can be found in the included studies cited in the references section. The PRISMA checklist and detailed data extraction forms are available from the corresponding author upon reasonable request.

## Supplementary Materials

The following supporting information can be downloaded at https://www.mdpi.com/article/10.3390/children13020305/s1, Table S1: Search Strategy.

## Abbreviations

The following abbreviations are used in this manuscript:

UPJ	Ureteropelvic Junction
UVJ	Ureterovesical Junction
POM	Primary Obstructive Megaureter
UTI	Urinary Tract Infection
