# Supplementary material for: Dual-Level Ureteral Obstruction in Children: A Systematic Review Highlighting Diagnostic Challenges and Optimal Surgical Strategy
Source: Children (Basel). 2026 Feb 22;13(2):305. doi: 10.3390/children13020305 (PMC12939143; doi:10.3390/children13020305)
Supplement: Supplementary file 1 [file children-13-00305-s001.zip › children-4135763-supplementary.pdf]

Title of the Review: **Dual-Level Ureteral Obstruction in Children: A Systematic Review Highlighting Diagnostic Challenges and Optimal Surgical Strategy**

Prepared by:
Olivia-Oana Stanciu
"Carol Davila" University of Medicine and Pharmacy
Email: olivia.stanciu@drd.umfcd.ro

Date
20 January 2026

PURPOSE OF THE SEARCH STRATEGY:
This document describes the complete electronic search strategy used to identify relevant studies for the systematic review. The aim was to capture all published reports of ipsilateral concomitant ureteropelvic junction (UPJ) and ureterovesical junction (UVJ) obstruction in pediatric patients. Given the rarity of this condition, the strategy was intentionally broad and incorporated controlled vocabulary and free-text terms.

ELECTRONIC DATABASES SEARCHED:
The following databases were searched from inception to the final search date (8 January 2026):
• MEDLINE (via PubMed)
• Embase
• Scopus
• Web of Science Core Collection
• Cochrane Library (CENTRAL)

Additional sources:
• Manual screening of reference lists of included studies
• Forward citation tracking
• Grey literature screening limited to reports with extractable case data

KEY CONCEPTS
• Ureteropelvic junction obstruction
• Ureterovesical junction obstruction / primary obstructive megaureter
• Ipsilateral or concomitant obstruction
• Pediatric population

CONTROLLED VOCABULARY TERMS:
MEDLINE (MeSH):
Ureteral Obstruction; Kidney Pelvis; Ureter; Hydronephrosis; Infant; Child; Adolescent

Embase (Emtree):
ureteropelvic junction obstruction; ureterovesical junction obstruction; megaureter; hydronephrosis; child

FREE-TEXT TERMS
"ureteropelvic junction obstruction", "UPJO", "PUJO", "pelviureteric junction obstruction",
"ureterovesical junction obstruction", "UVJO", "primary obstructive megaureter",
"double obstruction", "coexisting obstruction", "concurrent obstruction", "combined obstruction",
"ipsilateral obstruction", "pediatric", "paediatric", "child*", "infant*", "neonate*"

DATABASE-SPECIFIC SEARCH STRINGS

PubMed (MEDLINE):

("ureteropelvic junction obstruction"[tiab] OR "UPJ obstruction"[tiab] OR "UPJO"[tiab] OR "PUJO"[tiab] OR "pelviureteric junction obstruction"[tiab])
AND
("ureterovesical junction obstruction"[tiab] OR "vesicoureteral junction obstruction"[tiab] OR "UVJO"[tiab] OR "primary obstructive megaureter"[tiab])
AND
("ipsilateral"[tiab] OR "coexisting"[tiab] OR "concurrent"[tiab] OR "double obstruction"[tiab])
AND
(child[MeSH Terms] OR infant[MeSH Terms] OR adolescent[MeSH Terms] OR pediatric[tiab] OR paediatric[tiab] OR child*[tiab] OR infant*[tiab])

Embase:

('ureteropelvic junction obstruction'/exp OR UPJO:ti,ab OR PUJO:ti,ab)
AND
('ureterovesical junction obstruction'/exp OR 'primary obstructive megaureter':ti,ab OR UVJO:ti,ab)
AND
(ipsilateral:ti,ab OR coexisting:ti,ab OR concurrent:ti,ab OR 'double obstruction':ti,ab)
AND
(child/exp OR pediatric*:ti,ab OR paediatric*:ti,ab OR infant*:ti,ab)

Scopus:

TITLE-ABS-KEY("ureteropelvic junction obstruction" OR UPJO OR PUJO)
AND TITLE-ABS-KEY("ureterovesical junction obstruction" OR UVJO OR "primary obstructive megaureter")
AND TITLE-ABS-KEY(ipsilateral OR coexisting OR concurrent OR "double obstruction")
AND TITLE-ABS-KEY(pediatric OR paediatric OR child* OR infant*)

Web of Science:

TS=("ureteropelvic junction obstruction" OR UPJO OR PUJO)
AND TS=("ureterovesical junction obstruction" OR UVJO OR "primary obstructive megaureter")
AND TS=(ipsilateral OR coexisting OR concurrent OR "double obstruction")
AND TS=(pediatric OR paediatric OR child* OR infant*)

Cochrane CENTRAL:

(ureteropelvic OR UPJO) AND (ureterovesical OR UVJO OR megaureter) AND (child OR pediatric)

RECORD MANAGEMENT

All records were exported into reference management software and duplicates were removed prior to screening.

SCREENING PROCESS

Two reviewers independently screened titles and abstracts, assessed full texts for eligibility, and resolved disagreements by consensus.
